# Congenital Factor V deficiency: perioperative management (case report)

**DOI:** 10.11604/pamj.2020.36.46.23561

**Published:** 2020-05-29

**Authors:** Mohamed Anass Fehdi, Mohamed Lazraq, Sabah Benhamza, Abdelhak Bensaid, Youssef Miloudi, Najib El Harrar

**Affiliations:** 1 Anesthesia and Critical Care Department, 20^th^ August Hospital, University Hospital Ibn Rochd, Casablanca, Morocco

**Keywords:** Anesthesiology, perioperative management, congenital factor V deficiency, hematology

## Abstract

Factor V congenital deficiency is a rare hereditary disease, it exposes patients to hemorrhagic risk, with high morbi-mortality. Its management is a real challenge for practitioners. Perioperative management of patients with Factor V congenital deficiency needs anesthetists, hematologists and surgeons to work in close collaboration.

## Introduction

Factor V congenital deficiency is a rare hereditary factor deficiency, due to lack in plasmatic concentration of factor V. It is responsible for hemorrhages, going from mild to severe hemorrhagic symptoms. The perioperative management of patients with factor V deficiency is a real challenge for anesthetists, as the hemorrhagic risk is higher in these patients. We report the case of a patient with factor V deficiency, and our experience in the perioperative management of her cochlear implant surgery.

## Patient and observation

A 45 years old female patient, with history of bilateral deafness, secondary to bilateral chronic otitis media. The pose of cochlear implant under general anesthesia was indicated. Preanesthetic evaluation indicated a safe anesthesia with no risk of complications. Biological assessment has showed an abnormal hemostasis, with a very low prothrombin time (PT) at 15%, and a long activated Partial Thromboplastin Time (aPTT) at 50 seconds (for a normal aPTT at 28 seconds). In the first time we referred our patient for a hematological assessment. The screening of plasmatic coagulation factors, has objectified a very low Factor V level at 1% (normal range from 70% to 120%). The screening of circulating antibodies was negative. Members of family were also tested, and the patient´s brother was found to be carrying the same factor deficiency, which indicated its hereditary nature. Further interrogation of the patient has clarified the presence of menorrhagia episodes in the past. For perioperative management, and with the help of hematologists, fresh frozen plasma (FFP) transfusion was performed, 24 hours before surgery, in order to bring factor V level between 15% and 20%. Another transfusion one hour before surgery was necessary. The surgical procedure was performed with no complications, particularly hemorrhagic accidents. The surgeons judged the hemostasis as satisfying during surgery. The transfusion of fresh frozen plasma was maintained at D-1 after surgery, and after that a daily screening of PT and Factor V level helped us modulating the FFP transfusion. The patient´s postoperative recovery was without any complications. She was transferred to hematology ward for further follow-up and surveillance.

## Discussion

Factor V congenital deficiency is rare hereditary disease. Its transmission is autosomal recessive, secondary to mutation in F5 gene (1q23) [1]. The biological diagnosis is based on the low level of Factor V, long aPPT and low PT [1-3]. It is responsible for a hemorrhagic syndrome, of variable severity, with no correlation between the plasmatic concentration of factor V and the severity of hemorrhagic symptoms, in fact it is more related to the level of factor V in platelets [4]. Our patients had a very low factor V level, with mild symptoms. Due to the lack of concentrated factor V, the perioperative management of patients with congenital factor V deficiency, requires a transfusion protocol with fresh frozen plasma, aiming a factor V level between 15% and 20%, the hemostasis then is sufficient [1-3]. Platelets transfusion might be necessary in severe cases [2,3]. Although the hemorrhagic risk, the final outcome is often favorable, as long as the diagnosis is done early, and the perioperative management suitably [1,2,4].

## Conclusion

The perioperative management of patients with congenital factor V deficiency requires an effective collaboration between anesthetists, surgeons and hematologists. The preanethetic evaluation is a crucial moment that should be done thoroughly. A transfusion protocol should be carried out suitably to guarantee a good outcome in these patients.

## Competing interests

The authors declare no competing interests.
